# Hands-on training about data clustering with orange data mining toolbox

**DOI:** 10.1371/journal.pcbi.1012574

**Published:** 2024-12-18

**Authors:** Janez Demšar, Blaž Zupan

**Affiliations:** 1 Faculty of Computer and Information Science, University of Ljubljana, Ljubljana, Slovenia; 2 Department of Education, Innovation and Technology, Baylor College of Medicine, Houston, Texas, United States of America; SIB Swiss Institute of Bioinformatics, SWITZERLAND

## Abstract

Data clustering is a core data science approach widely used and referenced in the scientific literature. Its algorithms are often intuitive and can lead to exciting, insightful results that are easy to interpret. For these reasons, data clustering techniques could be the first method encountered in data science training. This paper proposes a hands-on approach to data clustering training suitable for introductory courses. The education approach features problem-based training that starts with the data and gradually introduces various data processing and analysis methods, illustrating them through visual representations of data and models. The proposed training is suitable for a general audience, does not require a background in statistics, mathematics, or computer science, and aims to engage the audience through practical examples, an exploratory approach to data analysis with visual analysis, experimentation, and a gentle learning curve. The manuscript details the pedagogical units of the training, motivates them through the sequence of methods introduced, and proposes data sets and data analysis workflows to be explored in the class.

## Author summary

The highest satisfaction for any instructor comes from an engaged audience, a motivated class that pays attention, and student questions that open up new venues for exploring the planned material. Any introduction to data science deserves such an audience, while the burden is on the instructor to prepare an exciting lesson that covers the planned material and keeps students engaged with just the right mix of theory and practice. We could think of no better topic to cover in this way than an introduction to machine learning and no better way to introduce this field than through data clustering. Of course, by including the necessary ingredients to assist instructors: use cases to explore, a visual analytics environment to use in the classroom, and a set of problems to intuitively introduce concepts ranging from data representation, similarity scoring, clustering methods, to evaluation and explanation of the resulting models. In the manuscript, we propose the ingredients of such training and offer them in a form ready to be explored by instructors in practical, hands-on courses.

## Introduction

Data clustering is a fundamental exploratory data analysis technique [1,2]: in 2023 alone, more than 250 000 papers cited in PubMed contained the word “clustering” or “cluster” in the abstract or title. It is, therefore, a prerogative of students of the natural sciences, as well as to all other students, to become familiar with data clustering techniques early in their education or careers, both to understand the many applications of clustering in research and literature and to recognize what clustering techniques offer in the analysis of one’s own data.

Fortunately, learning about clustering can have a gentle learning curve and be enjoyable for teachers and students. As suggested in this manuscript, we can couple training in clustering methods with techniques from visual analytics [3] and visual presentation of data and results [4]. Data clustering can be introduced intuitively without immediately venturing into mathematical or algorithmic details. Given interesting data sets, the training can be problem-based, engaging, and inspiring. Visual analytics tools have already been successfully used for training in clustering; a prominent example of such a tool EduClust [5], which which enhances understanding through interactive visualizations and animations of clustering algorithms.

We propose a set of pedagogical units for hands-on training in data clustering. By sharing our lecture plans and associated training materials (available at https://notes.biolab.si/books/plos/clustering), we aim to support trainers and lecturers with notes, data sets, data analysis workflows, ideas for visual analysis, and short videos illustrating the software’s utility. The training can deliver these pedagogical units in a compact, single classroom session, covering a wealth of data science material conceptually within 2 to 4 h.

### The software toolbox

Our training emphasizes hands-on problem solving and mini-challenges, harnessing the power of Orange Data Mining [6,7], a free, open source data science platform that integrates data visualization, interactive graphing, and machine learning. Unlike most major visual programming data science tools, which are typically commercial, Orange is free to use and has been in continuous development since 2000 [6]. With nearly one million lines of source code, it has a large user base and a comprehensive feature set.

Orange provides an intuitive drag-and-drop interface that allows users to easily construct data analysis workflows from interconnected components. Workflows in Orange are interpreted. A key advantage of this approach is that changes are immediately reflected in the results. This differs from other popular data mining frameworks, such as KNIME [8] and RapidMiner [9], which require execution to see updates. This real-time feedback makes Orange particularly suitable for teaching, as students can quickly experiment with different settings and see the effects immediately. Orange also provides fully interactive visualizations, allowing users to zoom, pan, and select parts of the visualizations for deeper exploration. This interactivity creates a more engaging learning experience and simplifies complex concepts. While other tools could be used to deliver similar training, Orange’s accessible interface and interactive features increase student engagement, reduce the learning curve, and provide a rich set of pre-built data sets and workflows that are practical for classroom use.

### Didactic methods

The data clustering training proposed here is problem-based and is aligned with the higher-order thinking skills emphasized in Bloom’s Taxonomy [10]. In all of the proposed pedagogical units, we start with the data, find an appropriate visualization that reveals the problem we are going to work on, ask the students to use the visualization to find an appropriate solution, try to formalize it into an algorithm or a scoring function, apply it within a software tool, and scrutinize the results, which are again represented graphically whenever possible. Rather than formally describing the data analysis procedure and then applying it to the data, we have designed the pedagogical units to allow students to discover the algorithms on their own, possibly leading to “a-ha” moments and fostering critical thinking, development of problem-solving skills, and supporting self-directed learning.

Challenging students with practical problems and having them find the solution and generalize it to a procedure or algorithm is at the core of problem-based learning [11]. In terms of Bloom’s Taxonomy [10,12], this addresses higher-order thinking skills, including the ability to

- analyze the problem and represent objects of interest with the data and find and explain clusters;
- synthesize information and resources to formulate solutions by combining data visualization and various computational techniques, explaining clusters, and using the results for decision-making;
- apply the acquired knowledge to new situations since clustering is general and can be applied to any problem where we can measure the distances between objects of interest;
- evaluate the feasibility and effectiveness of different solutions by comparing different similarity metrics and clustering techniques, and by qualitatively or quantitatively comparing the clustering results.

### Teaching approach

While one can implement the proposed pedagogical units through a frontal lecture, an alternative is training in a computer classroom where students independently perform data analysis. This hands-on approach is preferable when feasible but requires more time and instructor assistance. Typically, a senior instructor leads the course, presents the analysis on screen, and challenges students to explore and find solutions independently. If students are unfamiliar with data science or the software toolbox, additional teaching assistants may be needed to help students when they get stuck and to interact with the instructor upon noticing interesting challenges in students’ work.

## Pedagogical units

The hands-on training includes 4 principal and 1 optional unit. In the Hierarchical Clustering unit, trainers define distances, perform manual clustering on a small data set, and compare it to a computer-generated dendrogram. The Cluster Explanation unit focuses on understanding clusters based on their features. The Inliers and Outliers unit introduces silhouette scoring and distance estimation. Silhouette scoring aids in estimating clusters in the *k*-Means Clustering unit, which covers centroids, cluster membership, and the data partitioning algorithm. The optional unit provides advanced clustering algorithms and practical considerations. Each unit progressively deepens students’ understanding of data, distances, clustering techniques, and visual exploration.

### Hierarchical clustering

**Fig 1:**
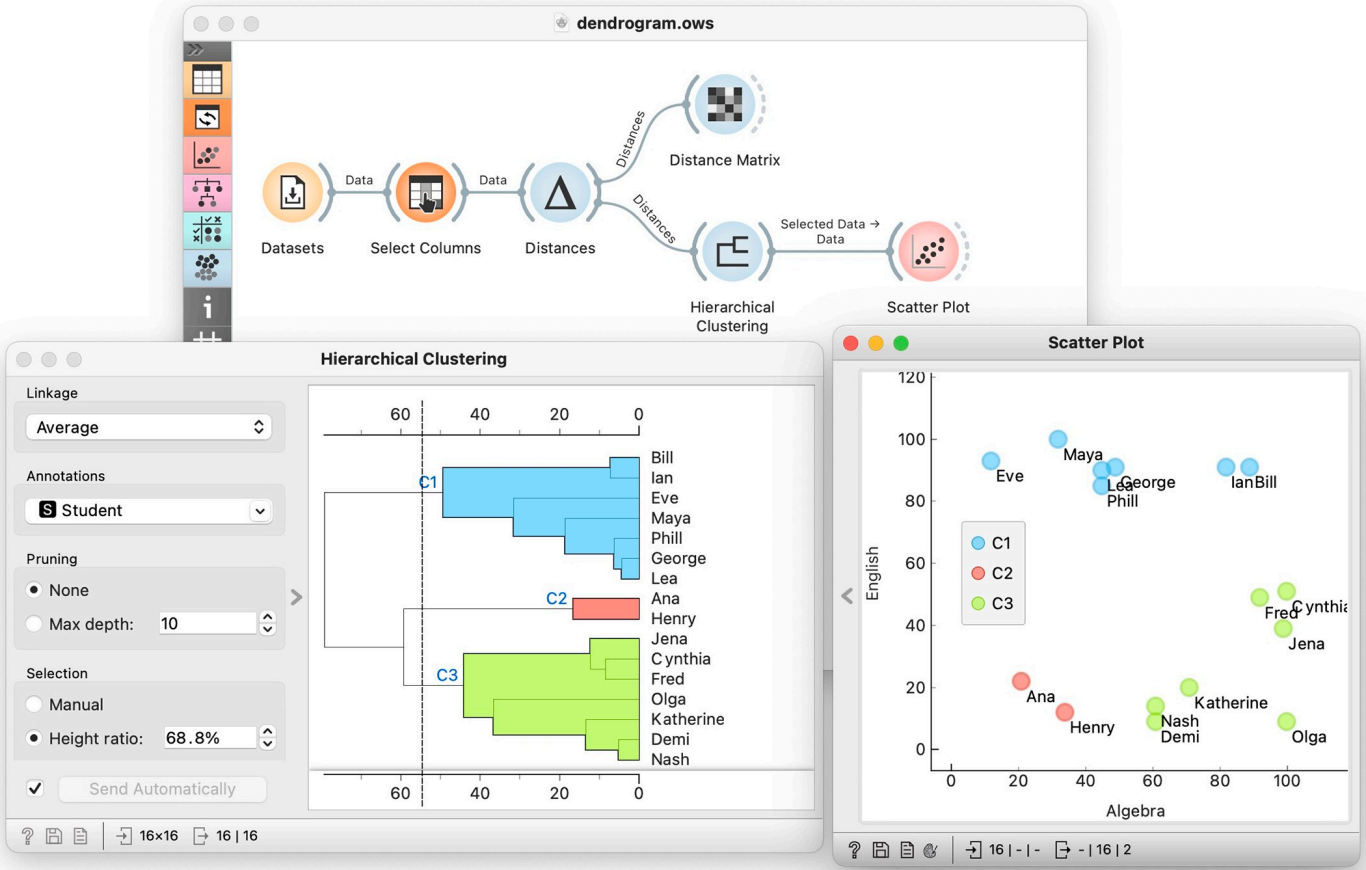
An Orange workflow we use in the early stages of training, where the student loads the data, selects 2 features, computes the distance matrix, then performs the clustering (Hierarchical Clustering) and displays the results in the scatter plot. The visualization of the dendrogram showing the result of the hierarchical clustering allows the user to interactively set the cut-off point, indicated by the vertical line; the cut-off point in the figure resulted in 3 clusters, which are also shown in the scatterplot.

Hierarchical clustering [13] is a popular clustering method with appealing visualization using dendrograms. In this unit, the trainers introduce the concepts of data clustering starting with a 2D data sets and extending these concepts to higher dimensions. Students learn about measuring distances between data points using Euclidean distance, estimating the distances between clusters, hierarchical clustering algorithm, and dendrograms to visualize clustering structure. They then explore the application of these concepts in multidimensional data spaces. The practical exercises with Orange reinforce these concepts through hands-on activities:

1. Start with a small data set comprising no more than 20 data instances described with a few features. Explain why inferring the groups of data instances for this problem domain would be beneficial.
2. Simplify the data set by selecting only 2 features and plotting the resulting data in the scatterplot.
3. Discuss possible groupings of data points in the scatterplot and have students suggest the number of clusters and their composition. Ask them what data instances make a good cluster. The discussion should lead to concepts of distance between data instances and its formalization in terms of Euclidean distance.
4. At this stage, it becomes clear that similar data instances, i.e., those within a small distance, should be in the same cluster. We introduce the idea of starting with individual data points, each forming its cluster, and iteratively merging the closest clusters.
5. It also becomes clear that we need to measure distances between emerging clusters consisting of multiple data instances. We ask students to suggest possible candidate definitions for what we will later call a *linkage function*.
6. Equipped with the means to measure distances between data points and clusters and with the concept of an agglomerative clustering procedure, we can now manually perform hierarchical clustering on our data set, on the blackboard, and present the result graphically, in a hand-drawn dendrogram.
7. Construct the dendrogram in Orange (Fig 1) and compare it with the one constructed by hand.
8. Now, use the data set with all features. Explain how to compute Euclidean distance in higher dimensional space. Explain that linkages used to estimate distances between clusters remain unchanged, as does the clustering procedure. Show the resulting dendrogram. Ask students what we should do next. Their answers should lead to the next lesson on explaining and understanding clusters.

### Cluster explanation

Characterizing the clusters by their defining characteristics is an important step in understanding the data. Considering that this may be the first time our trainees encounter the problem of cluster explanation, we are looking for the simplest means of cluster explanation that is still sufficient to provide interpretable results. Below, we propose the steps to introduce an explanation of hierarchical clustering while noting that we can use the described procedure with any type of clustering or to characterize any data subgroup:

1. Start with a simple multi-variate data set, possibly including at least 5 features, and construct hierarchical clustering. Use the resulting dendrogram to discuss the clustering structure and choose an interesting cluster for inspection.
2. Which features characterize our target cluster? We can use a box plot to find variables with different distributions of values within and without the target cluster.
3. Going manually through the variables and finding those that characterize the cluster works only for small data sets. We can use the Student’s *t* statistics to rank the variables according to their distribution in within-cluster and out-of-cluster data instances automatically.
4. At this stage, we are equipped for interactive cluster exploration and interpretation. In the dendrogram, we select a target cluster and then inspect *t* statistics-ranked variables that characterize the chosen cluster. Students can now reuse the approach on a more complex data set, again using the Orange interface.

Our accompanying lecture notes (see Software and Data Availability) propose sample data sets, Orange workflows, and combinations of visualizations to support this training plan. Although this unit covers an entirely new concept of explainability, it uses only one new component (the Box Plot) to achieve it, using a visual analysis and combinations of Orange components from the previous training unit.

### Inliers and outliers

Some data instances fit into their corresponding cluster better than others. We can easily spot the inliers and outliers in 2D data by observing them in a scatter plot. In higher dimensions, measuring how typical a data instance is for its own cluster requires a formal treatment. In this unit, we introduce one such measure, a silhouette score [14], which will later give rise to cluster quality scoring in the pedagogical unit on *k*-means clustering.

1. We start with a hand-drawn data set and discuss what makes a data item typical or atypical for a given cluster. In discussion with trainees, we reinvent a data item’s silhouette score.
2. A silhouette score borrows its name from the shape of its common visualization. We introduce silhouette scoring in Orange and verify its utility on a painted 2D data set.
3. We show that the concepts developed in 2 dimensions can be extended to multidimensional data sets and test the scoring on more complex data sets of choice.

This pedagogical unit (see “Inliers-and-Outliers” in the lecture notes referenced in Software and Data Availability) bridges the previous 2 and the next one by revisiting the concepts of distances and clusters, and (at this stage, intentionally implicitly) providing a tool to assess the quality of clustering. Namely, the silhouette scores are here introduced to spot the inliers and outliers, while it should be up to students to discover in the next unit that the mean silhouette could also be an estimate of the quality of the clustering.

### *k*-Means clustering

**Fig 2:**
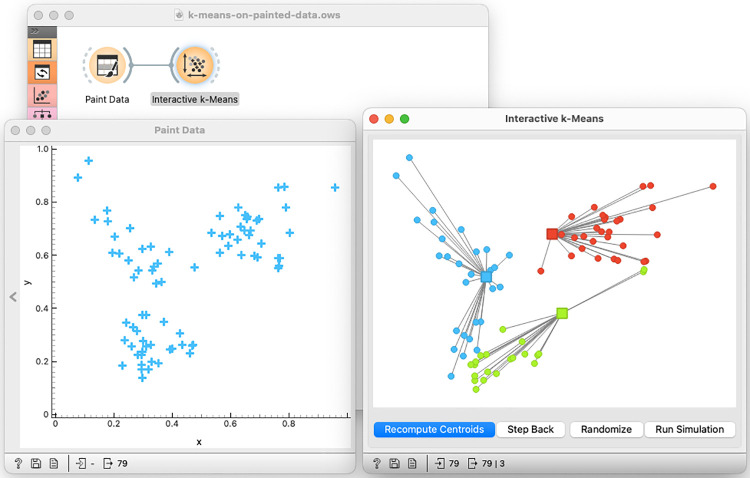
Interactive *k*-means in Orange. Several components of Orange were designed specially to support training in data science. In a shown workflow, trainees can paint the data and then in an interactive *k*-means clustering widget set the initial positions of centroids and execute the algorithms by, interchangeably, recomputing positions of centroids and reassigning centroid membership of data instances.

A popular alternative to hierarchical clustering is *k*-means clustering [15], a data partitioning approach and perhaps the most widely used clustering method in data science besides hierarchical clustering [16]. Pedagogically, it is interesting to introduce *k*-means clustering at this stage of training because it is a completely different clustering approach that lacks the visualization of a dendrogram, but offers speed gains and can handle larger data sets. In this pedagogical unit, we start with a painted data set to introduce the *k*-means algorithm, apply it to a multidimensional data set, and then reuse the clustering explanation techniques that students are already familiar with. The layout of the unit (see the chapter “K-Means Clustering” in the lecture notes) is as follows:

1. Motivate the use of *k*-means algorithm by exposing the potential weaknesses of hierarchical clustering.
2. Introduce the *k*-means algorithm on the painted data set. Explain the role of centroids (Fig 2).
3. Discuss the role of centroid initialization and show the cases where *k*-means fails due to improper initial placement of the centroids.
4. Construct the data and position the centroids so that *k*-means clustering would fail in finding obvious clusters. The aim here is to think about a proper centroid initialization procedure.
5. The number of clusters is an input parameter to *k*-means clustering. Could we guess the right number of clusters? Would a silhouette score help?
6. Equipped with the knowledge of the algorithm and procedure to estimate *k*, use *k*-means clustering on the multi-variate data set and explain the clusters.

### Common gotchas

**Fig 3:**
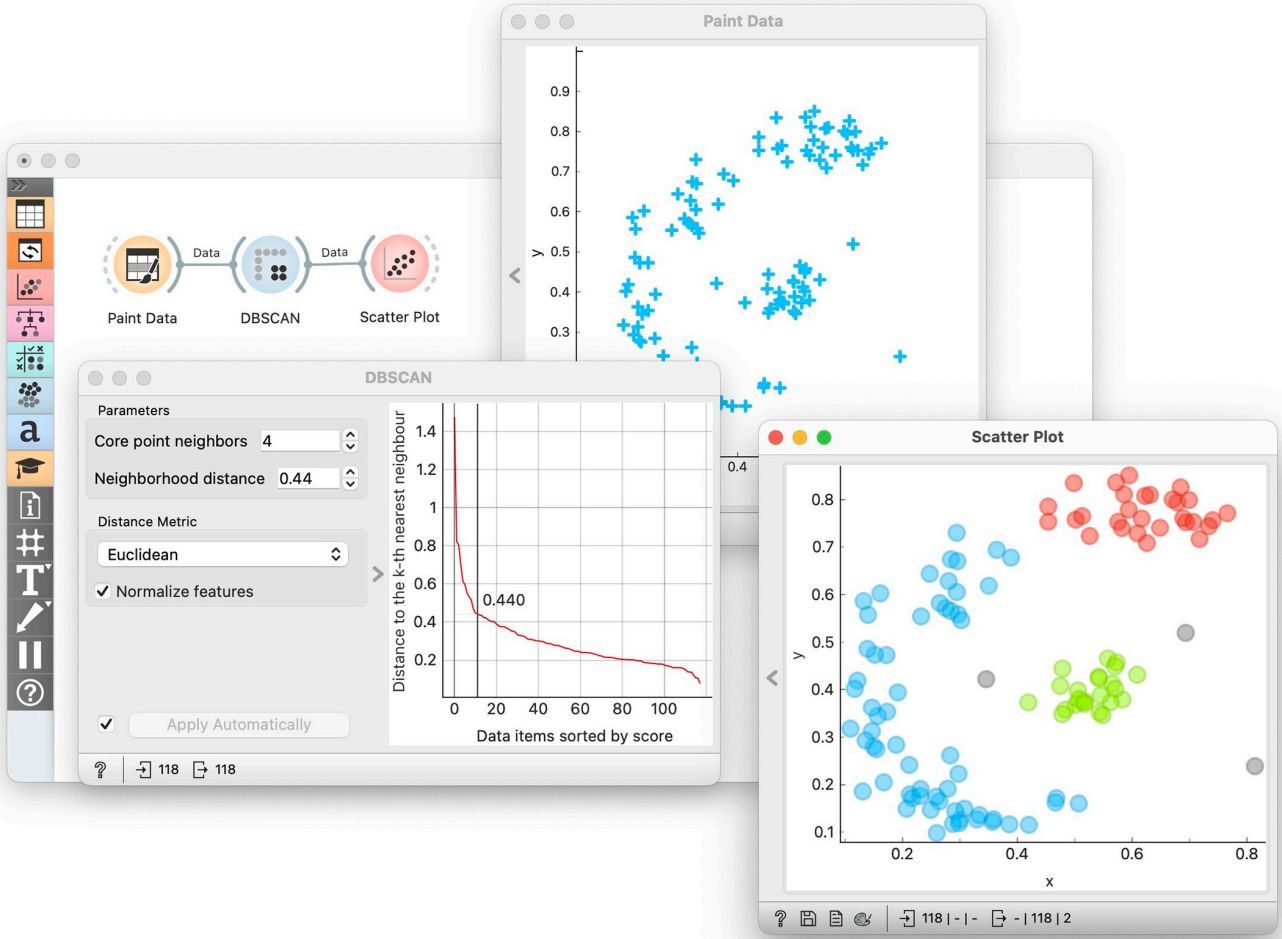
Experimenting with DBSCAN with a workflow where students draw the data and then interactively adjust the neighborhood distance parameter in DBSCAN’s scree plot, with the effects of this choice immediately visible in the scatter plot showing the clusters (in color) and outliers (in gray). DBSCAN is a clustering technique that is conceptually very different from hierarchical clustering and *k*-means, and as such it is a great candidate for a review in an additional pedagogical unit, time permitting.

In this optional unit (see “Common-Gotchas” in accompanying lecture notes), the trainers can highlight the limitations and assumptions of various clustering methods. Students may learn that dendrograms should not be used to infer pairwise differences between data instances and that k-means clustering assumes spherical clusters of comparable size, often failing with non-spherical or unequally sized clusters. They may also explore how clustering can sometimes identify clusters where none exist and understand the challenges in assessing clustering quality, including the use of silhouette plots. Finally, the unit touches on other clustering techniques such as DBSCAN [17] (Fig 3) and self-organizing maps [18], emphasizing their unique approaches and applications as well as the influence of parameter selection on results, as exemplified by the scree plot in DBSCAN.

## Discussion

Since 2015, we have been developing the proposed pedagogical units while training PhD students at the University of Ljubljana (Slovenia), Baylor College of Medicine (USA), and the Higher School of Economics (Moscow), refining our approach through hands-on master’s workshops at over 50 universities and conferences worldwide. We use Orange, a flexible data science toolbox, which has evolved based on our training experiences to include educational components, several designed specifically for clustering training. Orange has gained widespread adoption, being used as a primary training tool in machine learning classes at over 300 universities worldwide, according to a 2022 survey (https://orangedatamining.com/blog/orange-in-classroom-pt-2). This widespread classroom adoption has motivated us, the designers and project leads of Orange, to share our training experiences and the lecture plans and materials we have developed.

Teaching data science to biologists or any audience outside statistics, mathematics, or computer science is challenging due to their lack of background knowledge, time constraints, and the need for motivating examples. Our hands-on training addresses these issues with a concise set of lectures lasting no more than 3 h, focusing on intuitive understanding and gradually building knowledge. The training is practical, starting immediately with data exploration. Feedback from students and participants (not enclosed for privacy reasons) indicates that our approach quickly engages the audience and keeps them motivated through practical problems and experimentation.

Our pedagogical approach aims to cover many clustering concepts quickly, but this has its drawbacks. We focus on the use and features of clustering algorithms rather than their actual mechanics. Computer science students who implement clustering algorithms gain a deeper understanding of the topic. However, our training is designed for students outside computer science, leaving it up to them to explore further if they choose to after the course.

The pedagogical units proposed in this manuscript can be implemented in the early stages of data science or data literacy training, particularly in introductory courses. Their use of visual programming and analytics allows for covering a wide range of concepts quickly. These units can be followed by other machine learning topics, such as data projection and embedding, supervised modeling, explanatory AI, and model evaluation. For instance, we have recently proposed a similar hands-on approach for teaching overfitting [19]. The lectures outlined here may complement other data science training, serving as a precursor to more mathematically intensive courses that use Python or R for constructing data analysis pipelines.
